# Connecting the Past and Present: An Updated Literature Review of Aquagenic Syringeal Acrokeratoderma

**DOI:** 10.7759/cureus.76002

**Published:** 2024-12-19

**Authors:** Justin Lindsay, Aurelia R Incristi, Amelia Liu, Bryson Arnett, Marcelo Costa, Christian Chong

**Affiliations:** 1 Medicine, Wright State University Boonshoft School of Medicine, Dayton, USA; 2 Internal Medicine, Kettering Health Network, Kettering, USA

**Keywords:** aquagenic palmoplantar keratoderma, aquagenic pruritus, aquagenic syringeal acrokeratoderma, aquagenic wrinkling, aquagenic wrinkling of palms

## Abstract

Aquagenic syringeal acrokeradermatoma (ASA) is a dermatological condition characterized by the transient appearance of edematous, white, translucent papules on the palms, typically triggered by water exposure. While ASA is most commonly associated with cystic fibrosis (CF) and predominantly affects young females, there has been a significant increase in ASA cases since the most recent update in 2015. The COVID-19 pandemic increased the number of patients diagnosed with ASA following exposure to the viral infection. The growing body of literature suggests a multifactorial etiology for ASA, with potential links to CF, medication use, and possibly COVID-19-related behavioral changes. Due to the recent increase in cases of ASA, an updated review seeks to quantify the existing literature that has been published on the prevalence of this condition.

This review sought to find those newly diagnosed cases between the years 2014 and 2024. Through a literature review, we were able to find 57 cases of ASA since the last significant update to the total number of cases found in the literature. This review includes the prevalence of CF, a known etiology of ASA, as well as demographic information and known status of exposure to COVID-19.

## Introduction and background

Aquagenic syringeal acrokeradermatoma (ASA) is an acquired disease first described by English et al. as “transient reactive papulotranslucent acrokeratoderma” in 1996 [1-3]. Since then, the disease has had several names including aquagenic palmoplantar keratoderma, aquagenic keratoderma, aquagenic wrinkling of the palms, and ASA [3]. ASA is characterized by edematous white translucent papules and plaques that transiently localize on the palms, and less commonly, the soles, after immersion in water for three to five minutes [4-6]. The reaction lasts for between 20 and 30 minutes after removal from water immersion and drying [5,7]. Symptoms can also include tingling, pain, a sense of tightness, burning, or itching in the hands [4]. The diagnosis involves a combination of clinical presentation with transient reactive papulotranslucent acrokeratoderma following the “hands-in-the-bucket” technique and histopathology [2,8].

Historically, ASA has most commonly been presented in young females or those with cystic fibrosis (CF) [6,9]. However, new cases have recently been seen in males, those without a history of CF, and following the COVID-19 pandemic [2-4,6,8,9]. This underscores the potential underdiagnosis of ASA in populations not affected by CF.

Due to the rare nature of this disease, a clear understanding of the ASA prevalence is not yet fully known. A literature review was performed to solicit up-to-date numbers on the presence of ASA. While many different publications present a variety of different numbers for prevalence, we provide an up-to-date case count. In 2002, there were eight reported cases of ASA [10]. This number changed drastically when Ertürk-Özdemir et al. presented a case series of 10 patients diagnosed with ASA over a short period of 13 months, adding to the total of 35 cases previously recorded [6]. Their findings were significant, as this was the first case series to show male dominance. Since 2014, more cases have emerged, notably following the COVID-19 pandemic. A case series in 2021 reported 10 new cases occurring after the pandemic [7]. Subsequently, within the same year, a study was released relating six new cases of ASA to increased hand washing during the COVID-19 pandemic [11]. Furthermore, in 2022 eight new cases were found to occur days following a positive PCR test for COVID-19, suggesting a new link between ASA and COVID-19.

## Review

Methods

A literature review on ASA prevalence from 2014 to 2024 was conducted using electronic databases. This included PubMed and Web of Science, focusing on reported case studies and case series. Only articles published in English were considered for inclusion and that were published in the last 10 years. We used the following keywords: "ASA" and "Aquagenic Syringeal Acrokeratoderma". Terms were combined using Boolean operators (AND) in order to narrow the search results to ensure completeness.

Inclusion and exclusion criteria

Regarding inclusion and exclusion criteria, studies were selected if they had documented the (1) year, (2) gender of the patient, (3) age, (4) associated chronic conditions, and (5) COVID-19 status. Studies were excluded if there was (1) no available full-length, (2) the paper was redacted, and (3) outside the 2014-2024 time period.

Aim

The aim of this literature review is to compile new existing reports of ASA and provide an up-to-date total case count of this condition. The review intends to evaluate the demographics of the patients affected with ASA compared to the well-known presentations and associations of ASA.

Results

Study Selection and Data Analysis

**Figure 1:**
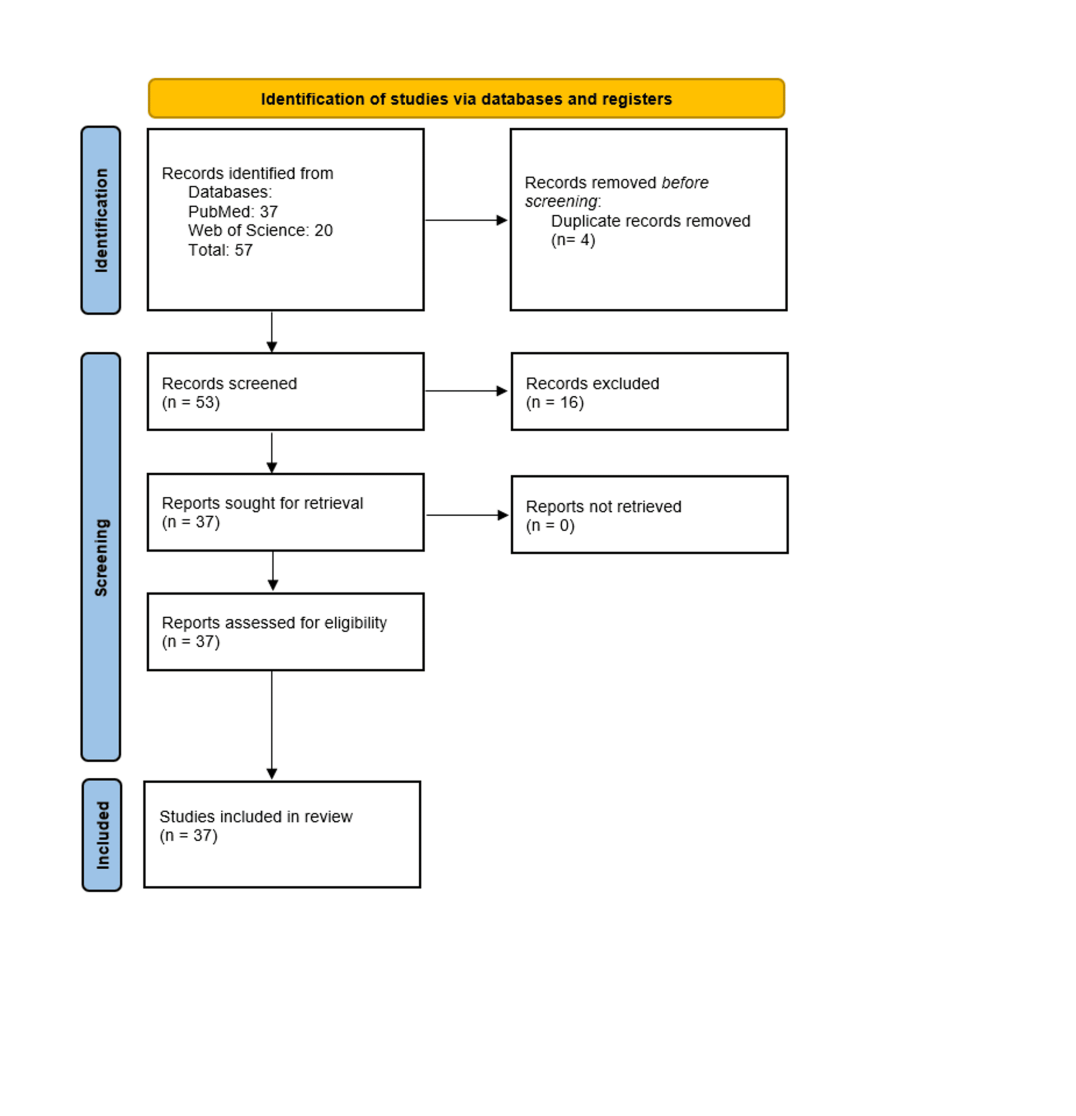
Preferred Reporting Items for Systematic Reviews and Meta-Analyses (PRISMA) Flow Diagram Case reports from 2014 to 2024 and their associated evidence and associations with ASA. The flowchart was created by the authors of the article with the help of empty flowchart obtained from an online source (PRISMA 2020 flow diagram. PRISMA statement. Accessed December 18, 2024. https://www.prisma-statement.org/prisma-2020-flow-diagram). ASA: aquagenic syringeal acrokeradermatoma

Cases reported from the years 2014-2024 were automatically included in the review process (Figure 1). This decision was based on a large past review paper published by Ertürk-Özdemir et al. that mentioned the total known case count of ASA was 35 cases. Beginning where past authors left off, we started with their totals and sought to add to this total while including their counts.

Figure 1 illustrates the selection process. Data collected from these studies were then analyzed by demographics, chronic conditions, clinical presentation, and COVID-19 status.

Study Characteristics

The characteristics of the included studies are displayed in Table 1. In this review, 37 studies comprised a case series, and isolated case reports were reported [2-9,11-39]. In total, our review revealed 77 new cases, of which 42 were female and 35 were male. All studies included were published between 2014 and 2024.

**Table 1:** Reported ASA Cases 2014-2024 [2-9,11-39] Summary of ASA literature review including demographic data, association with cystic fibrosis, COVID-19, and other chronic conditions. ASA: aquagenic syringeal acrokeradermatoma; PCOS: polycystic ovarian syndrome; HIV+: human immunodeficiency virus positive; CFTR: cystic fibrosis transmembrane conductance regulator; SC: stratum corneum; COVID-19: Coronavirus Disease 2019; n/a: not applicable; -: no prior medical history

Citation	Gender	Age	Chronic Conditions	History of Cystic Fibrosis?	Clinical Presentation	COVID-19 Status	Other Comments
Uyar [5]	Female	21	PCOS	No	Lesions on palms and soles	N/a	Associated with spironolactone
Kent et al. [14]	Female	19	-	No	Lesions on palms	N/a	
Sezer et al. [15]	Male	31	-	No	Lesions on palms	N/a	
Ghosh et al. [16]	Male	25	-	No	Lesions on palms and soles	N/a	
	Female	17	-	No	Lesions on palms and soles	N/a	
Ertürk-Özdemir et al. [6]	Female	12	Hyperhidrosis	No	White papules, edematous plaques, dilated eccrine ducts, exfoliation	N/a	
	Male	31	-	No		N/a	
	Male	26	Hyperhidrosis	No		N/a	
	Male	26	Hyperhidrosis	No		N/a	
	Male	26	-	No		N/a	
	Female	36	-	No		N/a	
	Female	20	Hyperhidrosis	No		N/a	
	Female	23	-	No		N/a	
	Male	22	Hyperhidrosis	No		N/a	
	Male	50	Hyperhidrosis	No		N/a	
Durmaz et al. [13]	Male	38	Hyperhidrosis	No	Translucent, whitish, pebbly papules on palms confined to central palmar creases	N/a	Successful treatment with calcipotriene 0.005%
	Female	25	Hyperhidrosis	No	Confluent, macerated, white, pavement stone-like papules w/ prominent puncta around the palmar flexures of both palms, assoc. w/ pruritus and tingling	N/a	
	Male	6 months	Hyperhidrosis	No	Whitish, cobblestone-like papules in palmar creases w/ profuse hyperhidrosis	N/a	
Kutlubay et al. [17]	Male	18	-	No	Bilateral swelling and whitish plaques on his palms and soles are presented.	N/a	
Dhawan et al. [18]	Female	35	-	No	Lesions on palms	N/a	
Angra et al. [19]	Female	13	-	No	Lesions on palms	N/a	
Fernandez-Crehuet et al. [20]	Female	22	-	No	Contact polarized manual dermoscopic examination was performed in all cases and evidenced the presence of well-defined ovoid yellowish structures located on the thenar and hypothenar areas of the palms.	N/a	
	Female	24	-	No		N/a	
	Female	50	-	No		N/a	
	Male	51	-	No		N/a	
Belli and Dogan [21]	Male	21	-	No	Whitening and swellings on the palms after every exposure to water for 2 weeks	N/a	
Lacarrubba et al. [22]	Female	19	-	yes	Lesions on palms	N/a	
Peña-Romero et al. [23]	Female	23	Multiple sclerosis	No	Palms: edema in superficial layers of the SC and acrosyringia, dilation of eccrine ducts	N/a	
	Female	19	No	No	Palms: edema in superficial layers of SC and acrosyringia, dilation of eccrine ducts	N/a	
	Female	6	No	No	Lesions on palms	N/a	
	Female	16	No	No	Lesions on palms	N/a	
	Male	65	No	No	Nose and upper lip: edema is superficial layers of SC and acrosyringia, dilation of eccrine ducts, thickened inner cuticle	N/a	
Zychowska et al. [24]	Male	13	-	No	Lesions on palms	N/a	
Cemil et al. [25]	Female	15	-	No	Lesions on palms	N/a	
Gürel et al. [26]	Female	15	-	No	Lesions on palms	N/a	
Chou et al. [27]	Male	58	Hyperhidrosis	No	Asymptomatic, whitish plaques on dorsal hands and wrists since childhood, apocrine metaplasia, no palmar involvement	N/a	
Okwunduet al. [28]	Female	22	-	No	Intense itching, white rugated plaques bilaterally on palms	N/a	
Montoya et al. [29]	Female	16	-	No	Multiple translucent papules on both palms, no other symptoms (burning, itching, etc.)	N/a	
Vazquez et al. [30]	Male	46	HIV +	No		N/a	
Narang et al. [31]	Female	31	-	No		N/a	
Kazandjieva et al. [32]	Female	7	-	No	Asymptomatic pinhead-sized whitish papule	N/a	
Liu et al. [33]	Male	41	-	No		N/a	Treated with 0% salicylic acid ointment once a day and 10% urea cream twice a day.
Rodríguez-Villa et al. [34]	Male	24	Hyperhidrosis	No	Mild hyperkeratosis on the palms and redundant skin on the knuckles in both hands	N/a	
Medhus et al. [35]	Female	30	-	No	Wrinkling and burning on palms, increased with the use of gloves	N/a	
Alay and Bilen [36]	Female	14	-	No		Positive	
Karagün [11]	Male	45	-	No	Increased hand-washing, whitish papules and plaques on palms burning	N/a	
	Male	33	-	No		N/a	
	Female	21	-	No		N/a	
	Male	52	-	No		N/a	
	Male	37	Hyperhidrosis	No		N/a	
	Male	41	-	No		N/a	
Ayhan et al. [7]	Female	24	-	No	All occurred after the COVID-19 pandemic, all localized to palms only	N/a	Siblings
	Male	22	-	No		N/a	
	Female	14	-	No		N/a	
	Male	6	-	No		N/a	
	Female	17	-	No		N/a	
	Female	13	-	No		N/a	
	Male	24	-	No		N/a	Siblings
	Female	17	-	No		N/a	
	Female	8	-	No		N/a	Siblings
	Male	5	-	No		N/a	
Burgos-Blasco et al. [12]	Female	5	-	No		Negative	All patients had resolution of disease within 2 months.
	Female	5	-	No		Positive	
	Male	8	-	No		Positive	
	Female	26	Hyperhidrosis	No		Positive	
	Female	30	-	No		Negative	
	Male	28	-	No		Negative	
	Male	32	-	No		Positive	
	Female	34	-	No		Positive	
Mahajan et al. [37]	Female	23	-	No	6‑8-month history of excessive wrinkling, swelling, and burning pain of palms within a few minutes of their coming in contact with water	N/a	
Marín-Hernández et al. [38]	Female	17	Chronic functional abdominal pain	No	2-month history of “wrinkling” of palms after contact with water. Palmar hyperlinearity and whitish, translucent papules forming plaques with a central depression were observed.	N/a	
Athira et al. [3]	Female	21	History of migraines, symptoms began 5 months after starting flunarizine	No	Papules localized to palmar creases specifically	N/a	
Demircioglu and Durmaz [9]	Male	4	-	No	Sloughing of skin occurred after pool time	N/a	
Weng et al. [2]	Male	40	History of erythema, pale brown flattened papules; family & childhood history of ichthyosis	No	Lesions present on dorsal hands, knees, elbows, buttocks, and ankles affected, sterile gloves and exhaustion worsened lesions.	N/a	Successful treatment with Botulism
Aguilera et al. [4]	Female	36	Contact dermatitis	No		N/a	
Dev and Verma [8]	Male	25	-	No	The case presented with features overlapping with hereditary papulotranslucent acrokeratoderma (HPA).	N/a	Treated with 20% aluminum chloride hexahydrate solution
Polascik et al. [39]	Male	24	Atopic dermatitis, food allergy anaphylaxis	No, negative for CFTR abnormalities	Bilateral palmar edema, whitish-colored papules, 3 months after COVID-19 infection	N/a	

Discussion

Previously, the majority of cases of ASA were diagnosed in young women [2,4,6,8,9]. Of the 77 new individual cases reported in this literature search, 42 (54.5%) individual cases were female and 35 (45.5%) individual cases were male. This showed a slight female predominance consistent with previous literature, but not to the extreme extent reported previously [2,4,6]. Furthermore, several case series demonstrated male dominance, with one study presenting six out of 10 cases as male, one showing two out of three cases as male, and a third case series reporting five out of six cases as male [6,11,13]. This suggests that the female gender may not be an essential contributing factor to the pathogenesis of the disease or that male cases were previously underdiagnosed. While many of the papers we found suggested male dominance, potential biases within case reports and case series, such as selection bias for unusual or interesting cases and reporting bias are an important influence on potential findings.

ASA is not a condition only diagnosed in young people, as the cases reported from 2014 to 2024 documented the youngest case at six months old and the oldest case at 65 years old [13,23]. The mean age of our review is 24.66, the median is 23 years, the mode is 24 years, and the range is 64.5 years. Additionally, while most cases have been acquired and sporadic, Ayhan et al. demonstrated a possible genetic tendency for the disease [7]. Of the 10 cases reported in the study, three pairs of siblings were diagnosed with ASA, with lesions appearing at the same time. Our review showed older men with uncommon presentations, including involvement of the nose, upper lip, wrists, knees, elbows, buttocks, and ankles. Interestingly, young adult populations of either gender demonstrated involvement of the soles in addition to their palms upon presentation. Individuals have demonstrated successful treatment with botulism and 0.005% calcipotriene. Other studies have reported several potential treatment options, such as topical aluminum chloride, glycerol, salicylic acid, urea and ammonium lactate creams, iontophoresis, and botulinum toxin [7]. There were eight cases that spontaneously resolved within 2 months of presentation, particularly following reduction with water contact. The variation in gender, age, treatment strategy, and potential genetic component necessitates further research into the true predisposing factors of ASA.

ASA was also thought to be predominantly associated with CF and is seen in up to 80% of CF patient's and up to 25% of carriers [40,41]. As of today, ASA is seen in up to 80% of CF patients and up to 25% of carriers [41]. It is thought that higher sodium concentrations from CF mediate increased water-binding capacity of keratins in the epidermis [42]. Interestingly, this literature search yielded only one case of ASA diagnosed in a 19-year-old female with CF, suggesting that while those with CF may be predisposed to the disease, the pathogenesis of ASA is not solely associated with CF [22].

A more modern potential association is with COVID-19 where 22 cases appeared in literature [7,11,12,36]. One suggested mechanism is consistent with increased hand-washing behavior or frequent contact with disinfectant [7,11,36]. While increased handwashing may trigger ASA, this does not explain the spontaneous resolution of patient symptoms following COVID-19 recovery [12]. Another case series of eight patients highlighted that the immunohistochemistry for the SAR-CoV-2 spike protein was positive in eccrine sweat glands, which could contribute to the dilation of the eccrine sweat glands commonly seen in ASA [5,12,43]. The histopathological studies from these patients also showed an abnormal perieccrine lymphocytic infiltrate forming blister-like patches on the palms [12]. Despite the increasing number of cases associated with COVID-19, it is fair to assume pathogenic mechanisms have remained consistent. Potential pathogenic mechanisms include increased sodium concentration in the skin, thus increasing the water retention in the stratum corneum, structural or functional defects in the stratum corneum, as well as aquaporin 3 channel dysregulation, and a role of weak eccrine duct walls [4].

The forenamed mechanisms can be explained histologically by the presence of ortho-hyperkeratosis with increased thickness and abnormal staining of the stratum corneum; dilated dermal eccrine ducts as well as hyperplasia of eccrine glands; increased capillaries around and adjacent to the eccrine glands [5,43]. This description is consistent with other reported cases where Dhawan et al. report compact orthokeratosis, hypergranulosis, and dilatation of intraepidermal eccrine ducts [18]. The dermis showed dilated blood vessels and numerous eccrine glands. While the presentation of ASA varies, it seems that there is a consistent histological display suggesting that despite variable inciting events or history the mechanism remains the same in all those with ASA.

## Conclusions

ASA is a condition with variable etiology and pathogenesis that leads to patient discomfort. Following our review of 77 cases, we added this to the previous 35 cases found by Ertürk-Özdemir et al. for a total of 112 cases. The recent increase in case reports involving men suggests that previous associations with women may not be entirely accurate. In addition, case reports included in this review from recent years showed one case of ASA in a patient with CF, which indicates that patients with CF may be predisposed to ASA, rather than strongly associated as previously assumed. The plethora of cases linked to COVID-19 may be explained by the hygienic practices used to prevent infection. These findings provide further nuance to the current understanding of ASA. Our review provides a new, up-to-date total for the overall prevalence of ASA in literature.
